# The Alkaloid Caulerpin Exhibits Potent and Selective Anti-Inflammatory Activity Through Interaction with the Glucocorticoid Receptor

**DOI:** 10.3390/md23060232

**Published:** 2025-05-29

**Authors:** Jônatas Sousa Pires dos Santos, Dahara Keyse Carvalho Silva, Vanessa da Silva Oliveira, Sergio Santos Silva Junior, Edivaldo dos Santos Rodrigues, Claudia Valeria Campos de Souza, Sabrina Teixeira Martinez, Osvaldo Andrade Santos-Filho, Cássio Santana Meira, Milena Botelho Pereira Soares

**Affiliations:** 1 Department of Life Sciences, State University of Bahia (UNEB), Salvador 40020-000, Brazil; 2 Gonçalo Moniz Institute, Oswaldo Cruz Foundation, FIOCRUZ, Salvador 40296-710, Brazil; 3 Laboratory of Molecular Modeling and Computational Structural Biology, Walter Mors Natural Products Research Institute, Health Sciences Center, Federal University of Rio de Janeiro (UFRJ), Rio de Janeiro 21941-599, Brazil; 4 Institute of Innovation in Advanced Health Systems (ISI SAS), University SENAI/CIMATEC, Salvador 41650-010, Brazil

**Keywords:** caulerpin, glucocorticoid receptor, macrophages, anti-inflammatory activity

## Abstract

Inflammation plays a central role in various pathological conditions, necessitating the search for safer and more effective anti-inflammatory agents. This study investigates the anti-inflammatory activity of caulerpin, a bisindolic alkaloid isolated from *Caulerpa racemosa*. In vitro assays demonstrated that caulerpin significantly reduced nitric oxide, TNF-α, IL-6, and IL-12 levels in macrophages stimulated with LPS + IFN-γ, without affecting cell viability. In silico toxicity predictions using Protox 3.0 reinforce a favorable safety profile of caulerpin. Molecular docking and molecular dynamics simulations revealed its high-affinity binding to the glucocorticoid receptor ligand-binding domain (GR-LBD), suggesting a mechanism of action similar to dexamethasone. The involvement of the glucocorticoid receptor was confirmed by the partial reversal of caulerpin’s effects upon RU486 treatment. In vivo, caulerpin exhibited a favorable safety profile, with no signs of acute toxicity at an oral dose of 100 mg/kg. Moreover, in a mouse model of endotoxic shock, caulerpin administration significantly improved survival rates in a dose-dependent manner, providing complete protection at 4 mg/kg. These findings highlight caulerpin as a promising candidate for the development of novel anti-inflammatory therapies. Further studies are warranted to explore its pharmacokinetics, optimize its structure, and evaluate its efficacy in chronic inflammatory diseases.

## 1. Introduction

Inflammation is a complex biological process triggered by the immune system in response to various stimuli, including tissue damage, infections, toxins, and physical or psychological stressors [1]. Its primary function is to restore homeostasis by eliminating the cause of cellular injury, removing damaged cells, and initiating tissue repair [2]. Inflammation is typically classified as either acute or chronic. Acute inflammation is a rapid and short-term response that resolves within minutes to days, whereas chronic inflammation arises from unresolved acute phases, persisting for months or years and contributing to the development of neoplasms, degenerative disorders, and organ dysfunction [3,4,5].

Although the treatment of inflammatory diseases often relies on nonsteroidal anti-inflammatory drugs (NSAIDs) and glucocorticoids, their prolonged use is associated with severe adverse effects, including gastrointestinal damage, renal impairment, cardiovascular complications, bone marrow suppression, and Cushing’s syndrome [6]. This highlights the need for new anti-inflammatory agents that offer both efficacy and improved safety profiles. In this context, caulerpin emerges as a promising alternative.

Caulerpin is a bisindolic alkaloid predominantly found in species of the genus *Caulerpa* (family Caulerpaceae), which includes approximately 97 species such as *C. cylindracea*, *C. mexicana*, *C. peltata*, and *C. racemosa* [7,8]. The edible green macroalga *Caulerpa racemosa* is traditionally harvested and cultivated in the Indo-Pacific region [9,10], and caulerpin is among its most abundant secondary metabolites [11,12]. This compound has been reported to exhibit a broad spectrum of biological activities, including anticancer, antitubercular, antioxidant, antidiabetic, and antiviral effects—particularly against SARS-CoV-2 [13,14,15]. Additionally, caulerpin displays notable anti-inflammatory properties, with experimental studies demonstrating its protective effects in murine models of peritonitis and colitis [16].

Although previous studies have reported the anti-inflammatory properties of caulerpin, important knowledge gaps remain regarding its safety profile and molecular mechanism of action. To the best of our knowledge, this is the first study to evaluate the acute toxicity of caulerpin and its therapeutic effects in a murine model of endotoxemic shock. Furthermore, this work provides novel evidence—supported by both in silico and in vitro approaches—implicating the glucocorticoid receptor (GR) as a key molecular target involved in caulerpin’s immunomodulatory activity. Therefore, this study aims to advance the pharmacological understanding of caulerpin and support its potential application in the treatment of inflammatory disorders.

## 2. Results and Discussion

**Figure 1:**
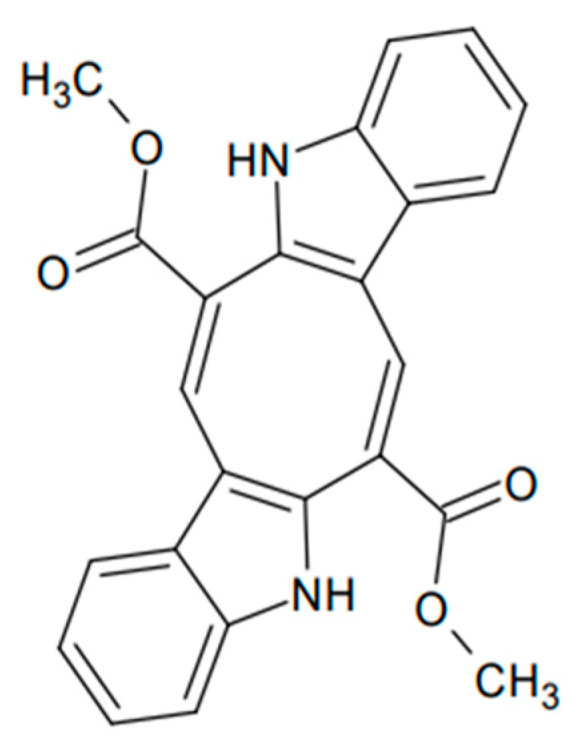
Chemical structure of caulerpin.

Initially, the in silico toxicity assessment of caulerpin (Figure 1) was performed across various organs and toxicological categories, using a 0.7 probability threshold to define significant predictions [17].

As shown in Table 1, caulerpin did not exhibit a significant probability for causing deleterious effects, such as hepatotoxicity, neurotoxicity, and nephrotoxicity, among others, suggesting a favorable safety profile in these aspects. Conversely, caulerpin demonstrated a high probability (>0.7) of being non-cardiotoxic, non-immunotoxic, and non-cytotoxic, further supporting its potential as a safe compound.

**Figure 2:**
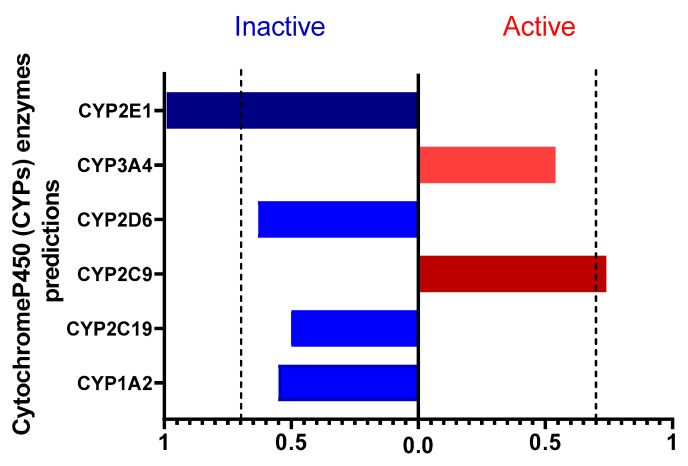
Prediction of caulerpin interaction with cytochrome P450 enzymes. The chart shows the enzymes predicted as active (red) or inactive (blue) in the metabolism of the compound. Values above 0.7 indicate that caulerpin may activate or inhibit certain CYP450 enzymes. A probability threshold of 0.7 was set for significance.

Using PROTOX 3.0, we also evaluated the interaction of caulerpin with cytochrome P450 (CYP450) enzymes (Figure 2). Among the analyzed CYPs, only CYP2E1 was predicted to be inactive; this enzyme primarily functions in the metabolism of xenobiotics. Caulerpin showed significant interaction with CYP2E1, an enzyme associated with the activation of pro-carcinogenic and hepatotoxic compounds. This suggests a lower risk of oxidative stress and liver injury, thereby supporting a favorable safety profile. Since CYP2E1 generates reactive oxygen species (ROS) that contribute to liver disease, its inactivity in response to caulerpin may reduce hepatic toxicity and the formation of toxic metabolites [18,19].

Caulerpin interacts with CYP2C9, a key enzyme involved in the metabolism of anticoagulants, NSAIDs, and oral hypoglycemics. This interaction suggests a favorable metabolic profile by enhancing drug biotransformation, reducing plasma concentrations, and minimizing adverse effects from drug accumulation [20]. CYP2C9 activation is particularly relevant for NSAID metabolism (e.g., ibuprofen, diclofenac, naproxen), potentially optimizing therapeutic efficacy while lowering gastrointestinal and renal risks [21,22]. Additionally, it may facilitate the formation of active metabolites with anti-inflammatory properties, thereby supporting the management of chronic inflammation and autoimmune diseases [23].

In addition to isoenzymes, PROTOX assesses the activation or inhibition of cellular receptors, transcription factors, and enzymes involved in metabolism, inflammation, hormonal modulation, and signaling pathways [24]. The heatmap in Figure S1 shows that caulerpin predominantly appears in the blue range, suggesting a favorable safety profile regarding toxic pathway activation. The inactivity of key biomarkers such as AhR [25], p53 [26], and PPAR-gamma [27] indicates a low potential for oxidative stress and cell cycle dysregulation, reducing carcinogenicity and cytotoxicity risks. Additionally, the low activity of ER and AR receptors suggests minimal endocrine interference [28]. Overall, these findings indicate a low toxicity profile, with few significant interactions with critical toxicity biomarkers, highlighting the need for further experimental validation to confirm caulerpin’s safety in therapeutic applications.

**Figure 3:**
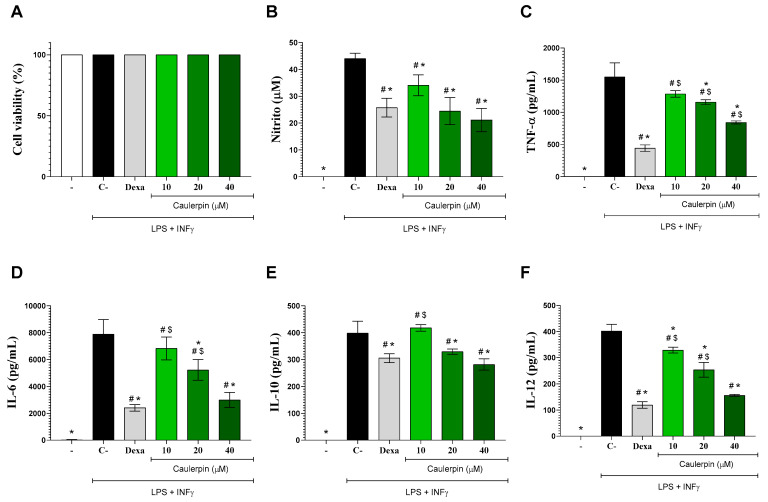
Effects of caulerpin on macrophages in vitro. Mouse peritoneal exudate macrophages stimulated or not with LPS + IFN-γ were cultured in the absence or presence of caulerpin (10, 20, or 40 µM) or dexamethasone (Dexa; 10 µM). (**A**) Cell viability was determined by the Alamar Blue method. Cell-free supernatants were collected for nitrite (**B**), TNF-α (**C**), IL-6 (**D**), IL-10 (**E**), and IL-12 (**F**) quantification. “−” refers to the group of untreated and unstimulated cells. C– refers to the group of untreated cells stimulated with LPS + IFN-γ. Data are expressed as the mean ± standard deviation (S.D.) of nine replicates obtained from three independent experiments. * *p* < 0.05 compared to stimulated and untreated cells; # *p* < 0.05 compared to unstimulated and untreated cells; $ *p* < 0.05 compared to dexamethasone-treated cells.

Regarding the biological results, initially, the cytotoxicity of caulerpin (10, 20, and 40 µM) was evaluated in peritoneal macrophage cultures. The compound showed no significant cytotoxicity at any of the tested concentrations, even in the presence of LPS and IFN-γ. Similar results were observed with dexamethasone, the standard drug used in immunomodulation assays (Figure 3A). Our findings align with those of Cuomo and colleagues (2021) [29], who also reported non-cytotoxic activity of caulerpin in gastric adenocarcinoma epithelial cells at concentrations up to 45 µM, with cell viability remaining above 80%. Likewise, Mert-Ozupek and collaborators (2022) [11] demonstrated that caulerpin does not exhibit cytotoxic effects on HDF (human dermal fibroblasts) and NIH-3T3 (mouse embryonic fibroblasts) cell lines.

The anti-inflammatory effects of caulerpin were subsequently assessed in macrophage cultures stimulated with LPS + IFN-γ. Nitrite levels were initially measured as an indicator of nitric oxide (NO) production. NO is a critical mediator of the inflammatory response, playing a key role in pathogen elimination by macrophages. However, excessive NO production can lead to cytotoxicity and tissue damage [30]. As shown in Figure 3B, LPS + IFN-γ stimulation significantly increased nitrite production (*p* < 0.05). However, treatment with caulerpin at concentrations of 20 and 40 µM resulted in a significant reduction in nitrite levels (*p* < 0.05), with inhibition rates of 44.5% and 52%, respectively. These findings align with those reported by Lee and colleagues (2012) [22], which demonstrated that fucoidan polysaccharides extracted from *Ecklonia cava* inhibited NO production in RAW 264.7 macrophages stimulated with lipopolysaccharide (LPS).

To further characterize the immunomodulatory effects of caulerpin, cytokine production by activated macrophages was quantified by ELISA. Stimulation with LPS + IFN-γ induced a marked increase in IL-6, IL-10, IL-12, and TNF-α levels (Figure 3C–F). However, treatment with caulerpin significantly reduced the production of these cytokines (*p* < 0.05). Under the same experimental conditions, dexamethasone (10 μM) also led to a significant decrease in cytokine levels. These findings are in agreement with those of Cuomo and colleagues (2021) [29], who showed that pre-treatment with caulerpin (15 μM) significantly downregulated the production of pro-inflammatory cytokines, including IL-1β, IL-6, IL-8, and TNF-α, by macrophages stimulated with *Helicobacter pylori*, a Gram-negative bacterium with an LPS-containing cell wall. These results are further supported by a study in infectious models, in which Sidrônio and colleagues (2025) [31] demonstrated that caulerpin at 25 and 50 μM significantly reduced IL-1β and TNF-α production in *RAW 264.7* macrophages infected with *Mycobacterium smegmatis* or *M. tuberculosis*. The authors also reported NLRP3 inflammasome modulation and the absence of cytotoxicity in Vero E6 and HepG2 cells, reinforcing the compound’s immunomodulatory potential and favorable safety profile across different cell systems.

Similarly, Bitencourt and colleagues (2015) [32] demonstrated the in vitro anti-inflammatory properties of aqueous and methanolic extracts from *Caulerpa mexicana*, which reduced IL-6, IL-12, and TNF-α production in peritoneal macrophages stimulated with LPS. In murine models of zymosan-induced peritonitis and DSS-induced ulcerative colitis, Lucena and colleagues (2018) [16] further reported that caulerpin treatment reduced TNF-α, IFN-γ, IL-17, and IL-6 levels while suppressing NF-κB transcription factor activity. Notably, caulerpin also increased IL-10 levels and reduced leukocyte migration into the peritoneal cavity. Consistently, Bitencourt and colleagues (2015) [32] demonstrated that methanolic extracts of *Caulerpa mexicana* also lowered IFN-γ, IL-6, IL-12, and TNF-α concentrations in colonic culture supernatants from mice with dextran sulfate sodium-induced colitis.

In order to investigate the mechanism of action of caulerpin, docking and molecular dynamics simulations were performed. In this study, the macromolecular target was the glucocorticoid receptor ligand-binding domain (GR-LBD).

**Figure 4:**
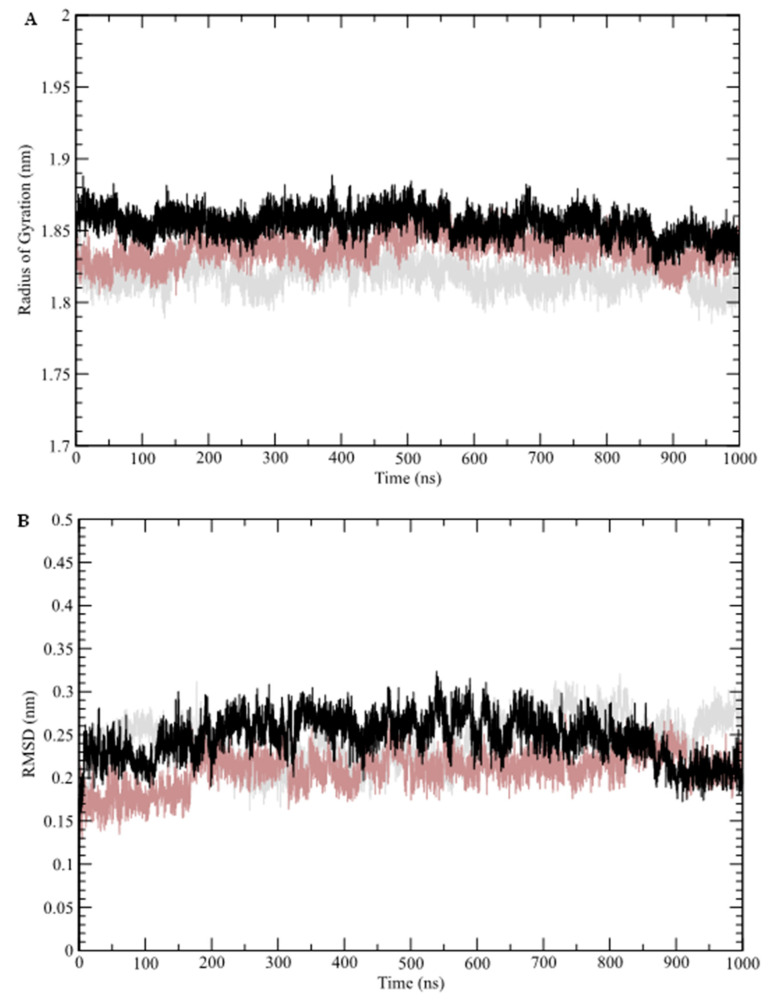
Molecular dynamics simulation analysis. (**A**) Radius of gyration (Rg); (**B**) root mean square deviation (RMSD). Molecular dynamics data for the free GR-LBD are represented by gray lines, the dexamethasone–GR-LBD complex by brown lines, and the caulerpin–GR-LBD complex by black lines.

The results of the molecular dynamics simulations conducted in this study revealed key structural insights. The radius of gyration (Rg) of the sampled structures was analyzed as a function of time (Figure 4A). Rg represents the mass distribution relative to the center of mass of the protein, providing insight into how well-packed (folded) the protein structure remains over time. As shown in Figure 4A, none of the systems exhibited significant deviations from their mean values: free GR-LBD (average Rg = 1.82 nm), caulerpin–GR-LBD complex (average Rg = 1.85 nm), and dexamethasone–GR-LBD complex (average Rg = 1.84 nm).

**Figure 5:**
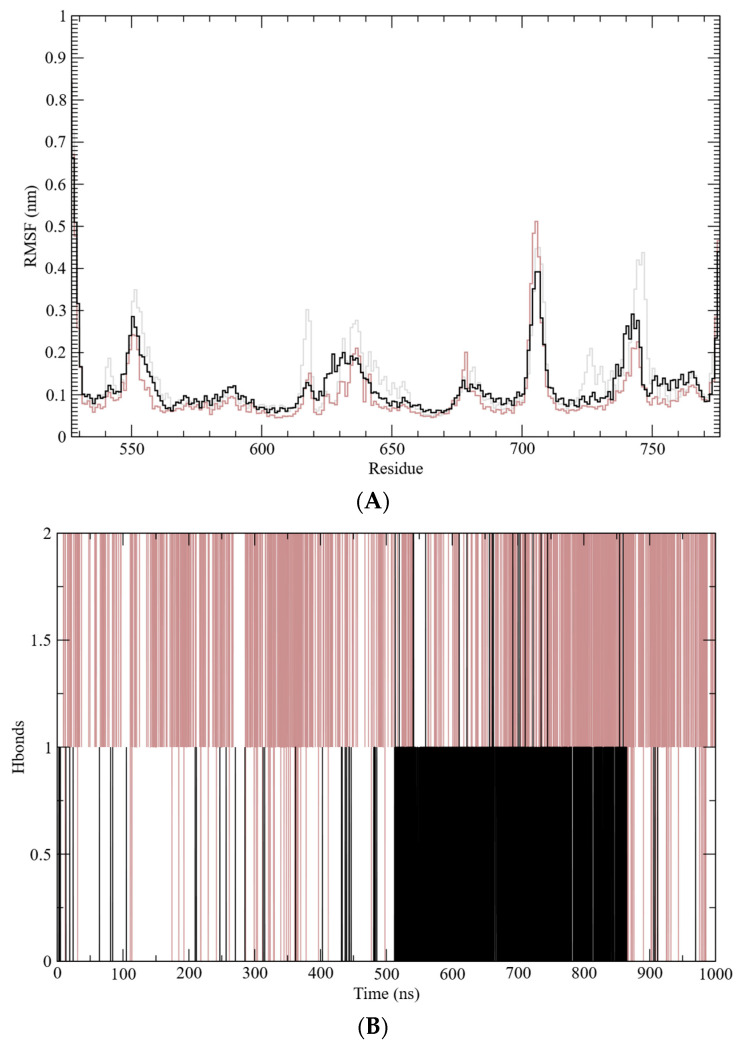
Molecular dynamics simulation analysis. (**A**) Root mean square fluctuation (RMSF); (**B**) hydrogen bond (H-bond) frequency. Molecular dynamics data for the free GR-LBD are represented by gray lines, the dexamethasone–GR-LBD complex by brown lines, and the caulerpin–GR-LBD complex by black lines.

The structural stability of the GR-LBD complexes was further assessed through root mean square deviation (RMSD) analysis, which monitors conformational changes over time (Figure 4B). This analysis indicates that the systems reached equilibrium after approximately 70 ns. The calculated average RMSD values for the free GR-LBD, dexamethasone–GR-LBD complex, and caulerpin–GR-LBD complex were 0.24 nm, 0.21 nm, and 0.25 nm, respectively. These results suggest that while both complexes exhibit structural stability, the dexamethasone–GR-LBD complex appears to be slightly more stable than the caulerpin–GR-LBD complex. Additionally, root mean square fluctuations (RMSF) were analyzed to evaluate residue-level flexibility by measuring the fluctuation of Cα atoms relative to the average structure sampled during the simulation (Figure 5A). The frequency of hydrogen bond formation along the simulation trajectory was also examined (Figure 5B). As shown, dexamethasone predominantly interacts with GR-LBD through at least two hydrogen bonds throughout the entire simulation, whereas caulerpin primarily engages with GR-LBD via a single hydrogen bond. Notably, between approximately 520 ns and 850 ns, the frequency of hydrogen bonds stabilizing caulerpin within the binding site increased significantly.

**Figure 6:**
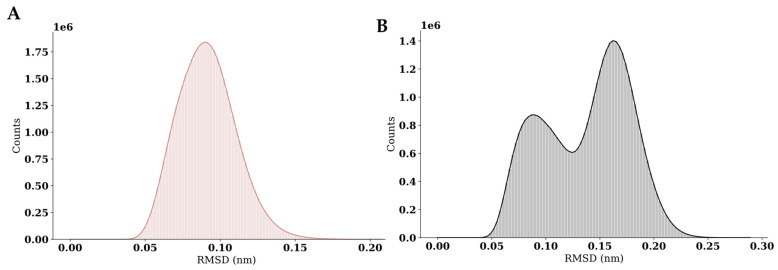
Cluster analysis of GR-LBD complexes with dexamethasone and caulerpin. (**A**) RMSD distribution for the dexamethasone–GR-LBD complex; (**B**) RMSD distribution for the caulerpin–GR-LBD complex.

**Figure 7:**
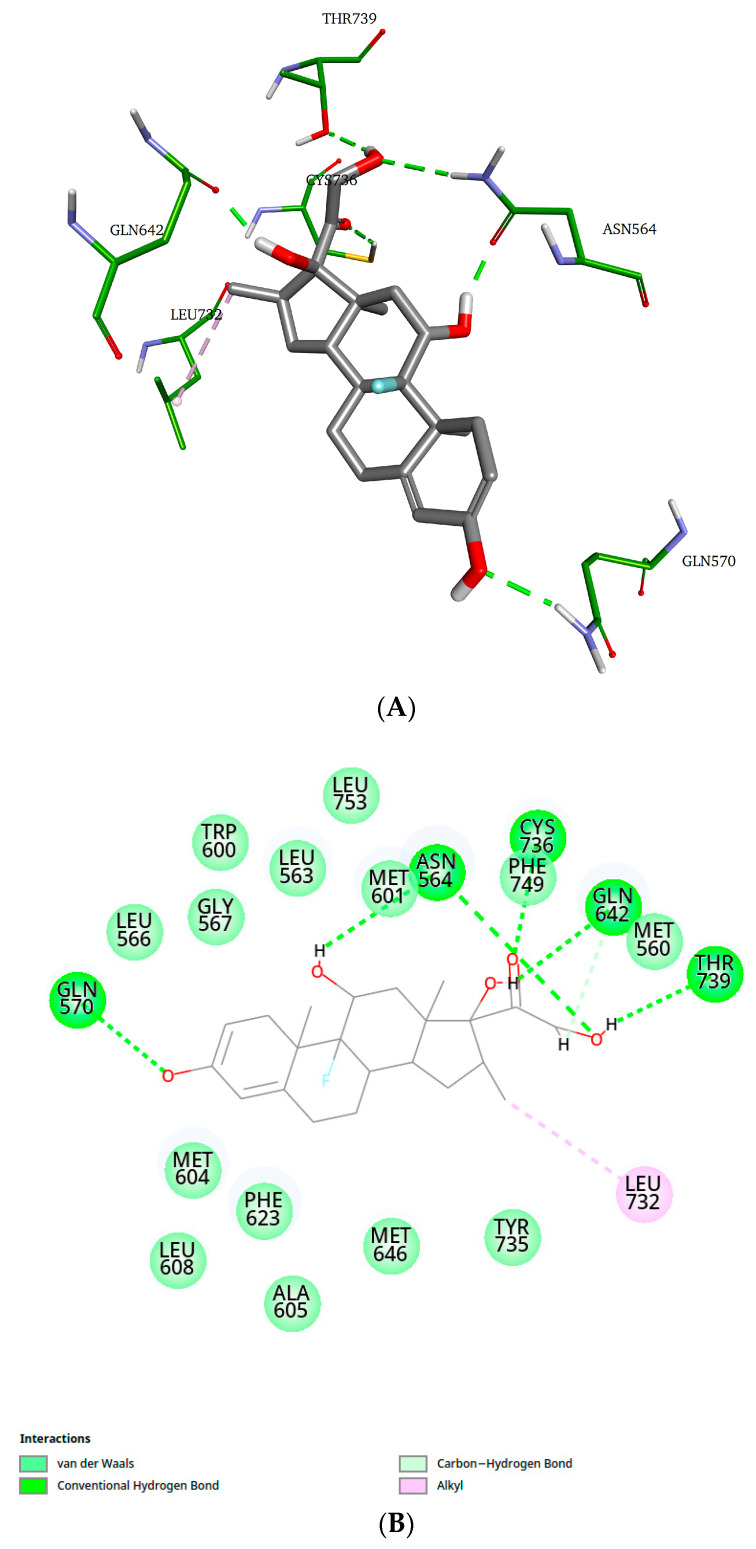
Representative structure of the dexamethasone–GR-LBD complex (cluster 1). (**A**) 3D representation; (**B**) 2D representation of dexamethasone–GR-LBD interactions.

To obtain representative ligand–GR-LBD interaction poses, a cluster analysis of the molecular dynamics trajectories was performed (Figure 6). A time window of 930 ns was used for pose sampling. The simulation of the dexamethasone–GR-LBD complex revealed a single dominant cluster, whereas the simulation of the caulerpin–GR-LBD complex identified two major clusters. These results suggest that caulerpin binds to the target protein binding site in more than one energetically favorable conformation, while dexamethasone adopts a single dominant binding mode. Representative intermolecular poses for the dexamethasone–GR-LBD and caulerpin–GR-LBD complexes are shown in Figure 6 and Figure 7, respectively.

As shown in Figure 7, dexamethasone interacts with residues Asn564, Gln570, Gln642, Cys736, and Thr739 through hydrogen bonds, and with residue Leu732 via an alkyl-type interaction. Additionally, the 2D diagram illustrates van der Waals interactions that are not represented in the 3D structure.

**Figure 8:**
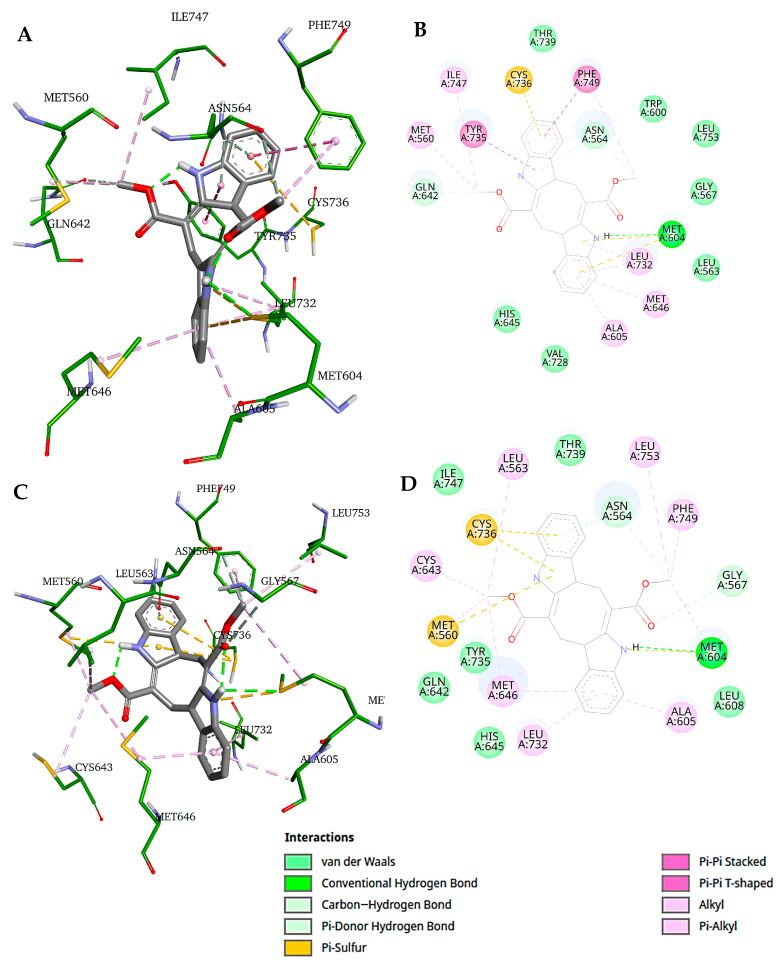
Representative structures of the caulerpin–GR-LBD complex (clusters 1 and 2). (**A**) 3D representation and (**B**) 2D representation of caulerpin–GR-LBD interactions for the representative structure from cluster 1; (**C**) 3D representation and (**D**) 2D representation of caulerpin–GR-LBD interactions for the representative structure from cluster 2.

Figure 8A,B illustrate the interaction of caulerpin with GR-LBD, as sampled from cluster 1 (Figure 6B). As shown, caulerpin forms a conventional hydrogen bond with Met604, a carbon–hydrogen bond with Asn564 and Gln642, and π–π stacked and T-shaped interactions with Phe749 and Tyr735, respectively. Additionally, it engages in π–alkyl interactions with Ala605, Met646, Leu732, and Phe749; alkyl interactions with Met560 and Ile747; π–sulfur interactions with Met604 and Cys736; and a π-donor hydrogen bond with Asn564. Van der Waals interactions are also depicted in the 2D diagram.

Figure 8C,D depict the intermolecular interactions sampled from cluster 2. In this conformation, caulerpin forms a conventional hydrogen bond with Met604 and carbon–hydrogen bonds with Asn564 and Gly567. It also interacts via π–alkyl interactions with Ala605, Met646, Leu732, and Phe749; alkyl interactions with Met560, Leu563, Cys643, Met646, and Leu753; and π–sulfur interactions with Met560 and Cys736. Van der Waals interactions are also represented in the 2D diagram.

Two key differences can be observed between the intermolecular poses sampled from clusters 1 and 2: (a) caulerpin does not form a π–sulfur interaction with Met560 in cluster 1, and (b) caulerpin does not establish π–π interactions with Phe749 and Tyr735 in cluster 2. Despite binding to the protein in a similar conformation in both clusters, caulerpin exhibits a different spatial orientation.

Throughout the molecular dynamics simulations, the binding free energies of caulerpin and dexamethasone to the GR-LBD binding site were calculated. Caulerpin exhibited an estimated binding free energy of −24.07 kcal/mol, whereas dexamethasone showed a binding free energy of −32.64 kcal/mol. These results indicate that both molecules have a significant affinity for the GR-LBD binding site, with dexamethasone displaying a stronger interaction.

**Figure 9:**
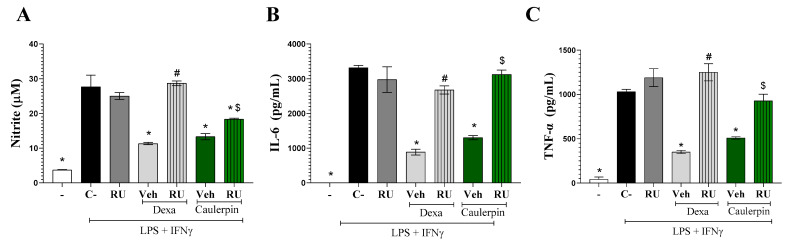
Involvement of glucocorticoid receptor in the immunomodulatory effect of caulerpin. (**A**) Nitrite concentrations, (**B**) IL-6 levels, and (**C**) TNF-α levels were measured in macrophages stimulated with LPS + IFN-γ. Cells were treated with caulerpin (40 µM) or dexamethasone (Dexa; 10 µM). In some cultures, cells were treated with these compounds in the presence of RU486 (glucocorticoid receptor antagonist, RU; 10 µM). “−” refers to the group of untreated and unstimulated cells. C– refers to the group of untreated cells stimulated with LPS + IFN-γ. Veh = Vehicle. Data are expressed as the mean ± S.D. of nine replicates obtained from three independent experiments. * *p* < 0.05 compared to stimulated and untreated cells; # *p* < 0.05 compared to stimulated and treated with dexamethasone-treated cells; $ *p* < 0.05 compared to caulerpin-treated cells.

To further investigate the role of glucocorticoid receptors in the immunomodulatory activity of caulerpin, the potential antagonistic effect of RU486 was assessed in stimulated macrophage cultures. The addition of RU486 (10 µM) to macrophage cultures stimulated with LPS + IFN-γ partially reversed the inhibitory effect of caulerpin (40 µM) on nitrite (Figure 9A) and completely reversed the production of IL-6 (Figure 9B) and TNF-α (Figure 9C). As expected, RU486 also blocked the inhibitory effect of dexamethasone (10 µM) on nitrite and cytokine levels, further supporting the involvement of glucocorticoid receptors in the anti-inflammatory activity of caulerpin.

Following the in vitro findings, we evaluated the potential toxicity of a single oral dose of caulerpin (100 mg/kg) in female BALB/c mice. Administration of caulerpin at this dose did not result in mortality or any observable signs of toxicity in the treated animals (Table 2). Additionally, no significant differences in body weight were observed between caulerpin-treated and vehicle-treated groups (Table 3). Consistent with our findings of no acute toxicity in mice, Russo and colleagues (2024) [33] reported that caulerpin did not induce toxicological alterations in *Mytilus galloprovincialis*, even when combined with caffeine. Notably, the study suggests a potential protective role of caulerpin against xenobiotic-induced toxicity, possibly via activation of peroxisome proliferator-activated receptors involved in detoxification pathways. These results reinforce caulerpin’s safety profile across different biological systems.

**Figure 10:**
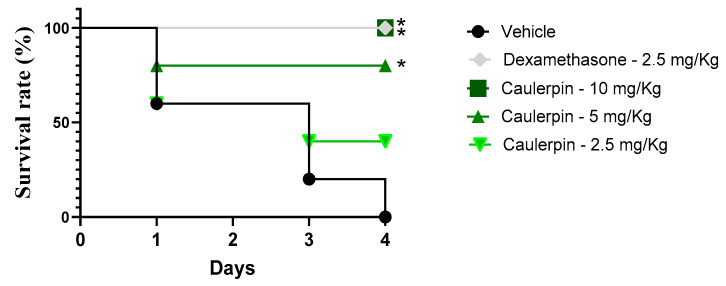
Survival curve of mice treated with caulerpin and subjected to endotoxic shock. Mice were orally treated with caulerpin (2.5, 5, or 10 mg/kg) or dexamethasone (2.5 mg/kg). Animals in the vehicle-treated group received saline solution containing 5% DMSO. Survival was monitored for four days following LPS challenge. Data represent results from two independent experiments. * *p* < 0.05, ** *p* < 0.01 compared to the vehicle-treated group. Statistical analysis was performed using the log-rank (Mantel–Cox) test.

Finally, the protective effect of caulerpin was evaluated in a murine model of endotoxic shock induced by a lethal dose of LPS. As shown in Figure 10, caulerpin treatment improved survival rates in a dose-dependent manner compared to the vehicle-treated group. Administration of 2.5 mg/kg resulted in a 40% survival rate, whereas the 5 mg/kg dose significantly extended survival (*p* < 0.05), reaching 80% survival by the end of the experiment. Notably, treatment with 10 mg/kg of caulerpin provided complete protection, ensuring 100% survival. Similarly, dexamethasone (2.5 mg/kg) also conferred full protection throughout the observation period.

The endotoxemic shock model is crucial for evaluating and confirming anti-inflammatory effects, particularly those targeting macrophages, as monocytes and macrophages are the primary sources of cytokines involved in sepsis and the resulting damage to target organs [34]. Thus, this finding reinforces previous results demonstrating the inhibition of key inflammatory mediators produced by macrophages.

Furthermore, other in vivo models involving caulerpin have been used in previous studies, supporting these findings related to sepsis. In the carrageenan-induced peritonitis model, Swiss albino mice treated with caulerpin were evaluated. Intraperitoneal pretreatment with caulerpin at a dose of 100 μmol/kg reduced leukocyte migration into the peritoneal cavity by approximately 48% [35].

Taken together, our results provide new insights into the pharmacological potential of caulerpin as a selective anti-inflammatory agent. Notably, the identification of the glucocorticoid receptor as a key molecular target represents a novel mechanistic contribution, differentiating our study from previous reports that merely described caulerpin’s anti-inflammatory effects without exploring its mode of action. While prior studies demonstrated the protective effects of caulerpin in murine models of colitis and peritonitis [16,35], none investigated its interaction with GR-LBD or employed molecular dynamics simulations and receptor antagonism assays (e.g., RU486) to confirm target engagement.

The dual binding modes observed in our cluster analysis suggest that caulerpin exhibits greater conformational flexibility than dexamethasone, which could be advantageous or detrimental depending on the biological context. This hypothesis warrants further investigation through crystallographic studies or extended MD simulations in different cellular environments.

Despite these promising findings, some limitations must be acknowledged. First, although RU486 experiments suggest GR involvement, definitive validation through genetic approaches (e.g., GR knockout or siRNA silencing) was not performed. Second, the in vivo efficacy of caulerpin was only evaluated in an acute model of endotoxic shock. Future studies should explore its effects in models of chronic inflammation, autoimmune diseases, or glucocorticoid resistance. Additionally, although in silico data point to a favorable safety profile, pharmacokinetic and metabolism studies are still necessary to assess oral bioavailability, half-life, and potential drug–drug interactions.

## 3. Materials and Methods

### 3.1. Drugs

Dexamethasone (Sigma-Aldrich, St. Louis, MO, USA), a synthetic glucocorticoid, was used as a positive control in immunomodulatory assays. Mifepristone (RU 486; Sigma-Aldrich), an antagonist of glucocorticoid receptors, was used in mechanism assays. Caulerpin was isolated from *Caulerpa racemosa* as previously described [36] (Figure 1). All compounds were initially solubilized in dimethyl sulfoxide (DMSO; Synth, São Paulo, SP, Brazil) and subsequently diluted in Dulbecco’s Modified Eagle Medium (DMEM; Life Technologies, Carlsbad, CA, USA) for in vitro assays. The final DMSO concentration was maintained below 0.1% in all experiments. For in vivo assays, compounds were prepared in a vehicle solution consisting of 5% DMSO and 95% saline.

### 3.2. Animals

BALB/c mice aged 4 to 8 weeks were obtained from the animal breeding facility of the Gonçalo Moniz Institute, Salvador, Brazil. Animals were housed in sterilized cages under controlled environmental conditions, including a temperature of 22 ± 2 °C and humidity of 55 ± 10%. They were provided water ad libitum and a nutritionally balanced rodent diet. All experimental procedures were reviewed and approved by the Institutional Committee on the Ethical Use of Laboratory Animals (approval number: L-IGM-019/24).

### 3.3. In Silico Toxicity Prediction of Caulerpin Using PROTOX 3.0

The toxicity profile of caulerpin was assessed in silico using the PROTOX 3.0 online tool (http://tox.charite.de/protox_II/; accessed on 30 August 2024). The chemical structure of caulerpin, defined by its SMILES notation (COC(=O)/C/1=C/C2=C(NC3=CC=CC=C23)/C(=C\C4=C1NC5=CC=CC=C45)/C(=O)OC), was entered into the platform to predict various toxicity parameters. These included acute toxicity classification, LD_50_ values, hepatotoxicity, immunotoxicity, mutagenicity, carcinogenicity, and cytotoxicity. Predictions were generated using the tool’s advanced machine learning models, trained on extensive toxicological datasets. The results provided insights into the potential safety profile of caulerpin, supporting subsequent experimental validation efforts.

### 3.4. Cytotoxicity Assay in Mammalian Cells

Peritoneal macrophages were isolated from BALB/c mice 4–5 days after intraperitoneal injection of 1.5 mL of 3% thioglycolate solution (Sigma-Aldrich) in saline. The harvested cells were seeded into 96-well plates at a density of 2 × 10^5^ cells/well in DMEM supplemented with 10% fetal bovine serum (FBS; GIBCO, Thermo Fisher Scientific, Waltham, MA, USA) and 50 µg/mL gentamicin (Life Technologies). The macrophages were incubated for 24 h at 37 °C in a humidified atmosphere containing 5% CO2_22. Subsequently, the cells were stimulated with LPS (500 ng/mL; Sigma-Aldrich) and IFN-γ (5 ng/mL; Sigma-Aldrich) and treated with caulerpin at concentrations of 10, 20, or 40 µM. After 72 h of incubation, 20 µL of Alamar Blue reagent (Invitrogen, Carlsbad, CA, USA) was added to each well, followed by an additional 4 h incubation. Colorimetric readings were performed using a SpectraMax 190 microplate reader (Molecular Devices, Sunnyvale, CA, USA). Three independent experiments were conducted, each performed in triplicate.

### 3.5. Macrophage Cultures

Peritoneal macrophages (2 × 10^5^ cells/well) were seeded into 96-well plates in DMEM supplemented with 10% FBS and 50 µg/mL gentamicin. Cells were incubated in triplicate under different conditions, including stimulation with LPS (500 ng/mL) and IFN-γ (5 ng/mL), and treated with caulerpin at various concentrations (10, 20, and 40 µM) or dexamethasone (10 µM). In some experiments, RU486 (10 µM), a glucocorticoid receptor antagonist, was added to the cultures to investigate the mechanism of action of caulerpin. After 4 h, cell-free supernatants were collected for TNF-α measurement, and after 24 h, supernatants were analyzed for IL-6, IL-10, IL-12, and nitrite levels. All samples were stored at −80 °C until analysis.

### 3.6. Cytokines and Nitric Oxide Production

Cytokine levels, including IL-6, IL-10, IL-12, and TNF-α, were measured in culture supernatants using enzyme-linked immunosorbent assay (ELISA) DuoSet kits (R&D Systems, Bio-Techne, Minneapolis, MN, USA), according to the manufacturer’s instructions. Nitric oxide production was quantified in macrophage supernatants using the Griess reaction, with nitrite concentrations serving as an indicator [37].

### 3.7. Acute Toxicity in Mice

Female BALB/c mice (6–8 weeks old) were randomly assigned to two groups and administered a single oral dose of caulerpin (100 mg/kg) or vehicle (5% DMSO in saline). Animals were observed daily for 14 days to assess clinical signs of toxicity, including alterations in the eyes, fur, and skin, as well as symptoms such as tremors, salivation, convulsions, diarrhea, lethargy, and coma. Body weights were recorded on days 0, 7, and 14 to monitor potential effects on growth and overall health [38].

### 3.8. LPS-Induced Endotoxin Shock

Male BALB/c mice (4 weeks old) were treated intraperitoneally with caulerpin (2.5, 5, or 10 mg/kg), dexamethasone (2 mg/kg), or vehicle. Ninety minutes after treatment, the mice were challenged intraperitoneally with 600 µg of lipopolysaccharide (LPS; serotype 0111:B4, *Escherichia coli*; Sigma-Aldrich) dissolved in saline. Survival rates were monitored daily over a period of 4 days.

### 3.9. Dexamethasone and Caulerpin Structures

The structure of caulerpin was obtained from the PubChem database under the identification code 5326018 [39]. The structure of dexamethasone, which was found co-crystallized within the glucocorticoid receptor (PDB ID: 1P93), was also used. Both ligands were energy-minimized using the DFT LC-BLYP/def2-TZVPP quantum method, implemented in the ORCA 5.0.4 package [40].

### 3.10. Molecular Docking

Molecular docking simulation was performed with GOLD 2024.2.0, a protein–ligand docking software based on a genetic algorithm (GA) [41]. Considering the GR-LBD binding site, a sphere with a radius equal to 10 Å was defined, with the geometric center coordinates set as: x = 45.320 Å; y = 12.050 Å; and z = 17.100 Å. The scoring function used was ChemPLP [42]. Default GA parameters GOLD were applied for sampling the docked poses.

### 3.11. Dynamics Simulations

Molecular dynamics simulations were carried out using GROMACS 2024.2 software for the free GR-LBD structure, as well as for the dexamethasone–GR-LBD and caulerpin–GR-LBD complexes [43]. The CHARMM36 force field was used [44], and ligand parameterization was performed using the CHARMM General Force Field (CGenFF) method [45]. A dodecahedral simulation box was created using the TIP3P water model, and sodium ions were added to neutralize the systems electronically. Periodic boundary conditions were applied. Each system was subjected to energy minimization using the steepest descent algorithm, with 50,000 steps and a convergence criterion of less than 2.39 kcal/mol. Subsequently, two 100 ps equilibration simulations were performed for both the free protein and the protein–ligand complexes. The first simulation used the NVT ensemble, followed by the NPT ensemble. In both cases, the temperature was maintained at 300 K. During the NPT simulation, pressure was kept constant at 1 bar. After equilibration, 1 μs (1000 ns) production molecular dynamics simulations were performed to calculate the binding free energies of each ligand with GR-LBD. Simulations were run under the NPT ensemble, with temperature control using the V-rescale implementation of Berendsen’s thermostat [46] and pressure control using the Parrinello–Rahman barostat [47]. The particle mesh Ewald (PME) method was used for long-range electrostatics [48]. Trajectory frames were sampled every 100 ps.

For binding free energy estimation, the time interval from 70 ns to 1000 ns was selected. Calculations were performed using the MM/PBSA method implemented in the gmx_MMPBSA tool [49]. For intermolecular interaction analysis, structural clustering was performed using the GROMOS method implemented in GROMACS, with an RMSD cutoff of 0.1 nm to group similar structures [50].

### 3.12. Visualization Tools and Plots

Molecular visualization was performed using BIOVIA Discovery Studio 2021 [51], and graphical analyses were conducted using the Grace plotting tool [52].

### 3.13. Statistical Analysis

The significance of differences between groups was evaluated using one-way ANOVA, followed by the Newman–Keuls multiple comparison post-test. Analyses were performed using GraphPad Prism version 8.0 (GraphPad Software, San Diego, CA, USA). Results were considered statistically significant when *p*-values were less than 0.05 (*p* < 0.05).

## 4. Conclusions

In this study, we demonstrated that caulerpin, a bisindolic alkaloid isolated from *Caulerpa racemosa*, exhibits potent and selective anti-inflammatory activity. In vitro, it significantly reduced nitric oxide, TNF-α, IL-6, and IL-12 levels in LPS + IFN-γ-stimulated macrophages without affecting cell viability. In silico toxicity predictions using PROTOX 3.0 support a favorable safety profile for caulerpin. Its mechanism of action involves high-affinity binding to the glucocorticoid receptor ligand-binding domain (GR-LBD), as revealed by molecular docking and dynamics simulations. This was corroborated by the partial reversal of its activity upon treatment with RU486, a glucocorticoid receptor antagonist. In vivo, caulerpin also exhibited a favorable safety profile, with no signs of acute toxicity at an oral dose of 100 mg/kg. Furthermore, caulerpin administration significantly improved survival rates in a murine model of endotoxic shock, providing complete protection at 4 mg/kg, further supporting its therapeutic potential in inflammatory conditions. Taken together, these findings position caulerpin as a promising candidate for the development of novel anti-inflammatory therapies, warranting further investigation into its pharmacokinetics, structural optimization, and efficacy in chronic inflammatory diseases.

## Figures and Tables

**Table 1:** Predicted toxicity of caulerpin across different organs and biological categories, along with the associated probability for each prediction.

Organ Toxicity or Toxicity End Points	Prediction	Probability
Hepatotoxicity	Inactive	0.59
Neurotoxicity	Inactive	0.7
Nephrotoxicity	Active	0.56
Respiratory toxicity	Active	0.5
Cardiotoxicity	Inactive	0.84
Carcinogenicity	Inactive	0.6
Immunotoxicity	Inactive	0.96
Mutagenicity	Active	0.53
Cytotoxicity	Inactive	0.71
BBB-barrier	Active	0.67
Ecotoxicity	Inactive	0.57
Clinical toxicity	Inactive	0.52
Nutritional toxicity	Inactive	0.52

A probability greater than 0.7 was used as the cutoff point to determine significant predictions.

**Table 2:** Effect of caulerpin on behavioral and general appearance of female BALB/c mice.

Behavior and General Appearance	Observations *	
	Vehicle	Caulerpin (100 mg/Kg)
Changes in the eyes	No changes	No changes
Changes in the fur	No changes	No changes
Changes in the skin	No changes	No changes
Coma	Absent	Absent
Convulsions	Absent	Absent
Diarrhea	Absent	Absent
Lethargy	Absent	Absent
Salivation	Absent	Absent
Sleep	Usual	Usual
Tremors	Absent	Absent

* Mice were observed daily for 14 days.

**Table 3:** Body weight of female BALB/c mice treated with caulerpin.

Days	Vehicle	Caulerpin (100 mg/Kg)
0	20.2 (± 1.0)	19.5 (± 1.9)
7	22.2 (± 0.8)	22.7 (± 1.0)
14	24.2 (± 1.1)	24.4 (± 0.8)

Values represent the mean ± standard deviation of six animals per group.

## Data Availability

The data presented in this study are available on request from the corresponding author.

## Supplementary Materials

The following Supporting Information can be downloaded at: https://www.mdpi.com/article/10.3390/md23060232/s1, Figure S1: Heat map representing the activity of different biomarkers for toxicity pathways.

## Abbreviations

BBB	blood–brain barrier
CYP	cytochrome P450
CGenFF	CHARMM General Force Field
DFT	density functional theory
Dexa	dexamethasone
DMEM	Dulbecco’s Modified Eagle Medium
DMSO	dimethyl sulfoxide
DSS	dextran sulfate sodium
ELISA	enzyme-linked immunosorbent assay
FBS	fetal bovine serum
GA	genetic algorithm
GR	glucocorticoid receptor
GR-LBD	glucocorticoid receptor ligand-binding domain
IFN-γ	interferon gamma
IL	interleukin
LD50	lethal dose 50%
LPS	lipopolysaccharide
MD	molecular dynamics
MM/PBSA	molecular mechanics/Poisson–Boltzmann surface area
NF-κB	nuclear factor kappa B
NO	nitric oxide
NSAIDs	nonsteroidal anti-inflammatory drug
NVT	constant number of particles, volume, and temperature ensemble
NPT	constant number of particles, pressure, and temperature ensemble
ORP	oxidation-reduction potential
PBMC	peripheral blood mononuclear cells
PME	particle mesh Ewald
PROTOX	in silico toxicity prediction tool
PPAR-γ	peroxisome proliferator-activated receptor gamma
RMSD	root mean square deviation
RMSF	root mean square fluctuation
ROS	reactive oxygen species
TNF-α	tumor necrosis factor alpha
