# Supplementary material for: The Alkaloid Caulerpin Exhibits Potent and Selective Anti-Inflammatory Activity Through Interaction with the Glucocorticoid Receptor
Source: Mar Drugs. 2025 May 29;23(6):232. doi: 10.3390/md23060232 (PMC12194734; doi:10.3390/md23060232)
Supplement: Supplementary file 1 [file marinedrugs-23-00232-s001.zip › marinedrugs-3651410-supplementary.pdf]

Supplementary Information

The Alkaloid Caulerpin Exhibits Potent and Selective Anti-inflammatory Activity Through Interaction with the Glucocorticoid Receptor

Jônatas Sousa Pires dos Santos[1], Dahara Keyse Carvalho Silva[2], Vanessa da Silva Oliveira[1], Sergio Santos Silva Junior[2], Edivaldo dos Santos Rodrigues[3], Claudia Valeria Campos de Souza[3], Sabrina Teixeira Martinez[4], Osvaldo Andrade Santos-Filho[3], Cássio Santana Meira[1,2,4,*], Milena Botelho Pereira Soares[2,4],*

[1] Department of Life Sciences, State University of Bahia (UNEB), Salvador 40020-000, BA, Brazil;

[2] Gonçalo Moniz Institute, Oswaldo Cruz Foundation, FIOCRUZ, Salvador 40296-710, BA, Brazil;

[3] Laboratory of Molecular Modeling and Computational Structural Biology, Walter Mors Natural Products Research Institute, Health Sciences Center, Federal University of Rio de Janeiro (UFRJ), Rio de Janeiro 21941-599, RJ, Brazil;

[4] Institute of Innovation in Advanced Health Systems (ISI SAS), University SENAI/CIMATEC, Salvador 41650-010, BA, Brazil.

*Correspondence: cassio.meira@fieb.org.br; milena.soares@fiocruz.br

Supplementary Information

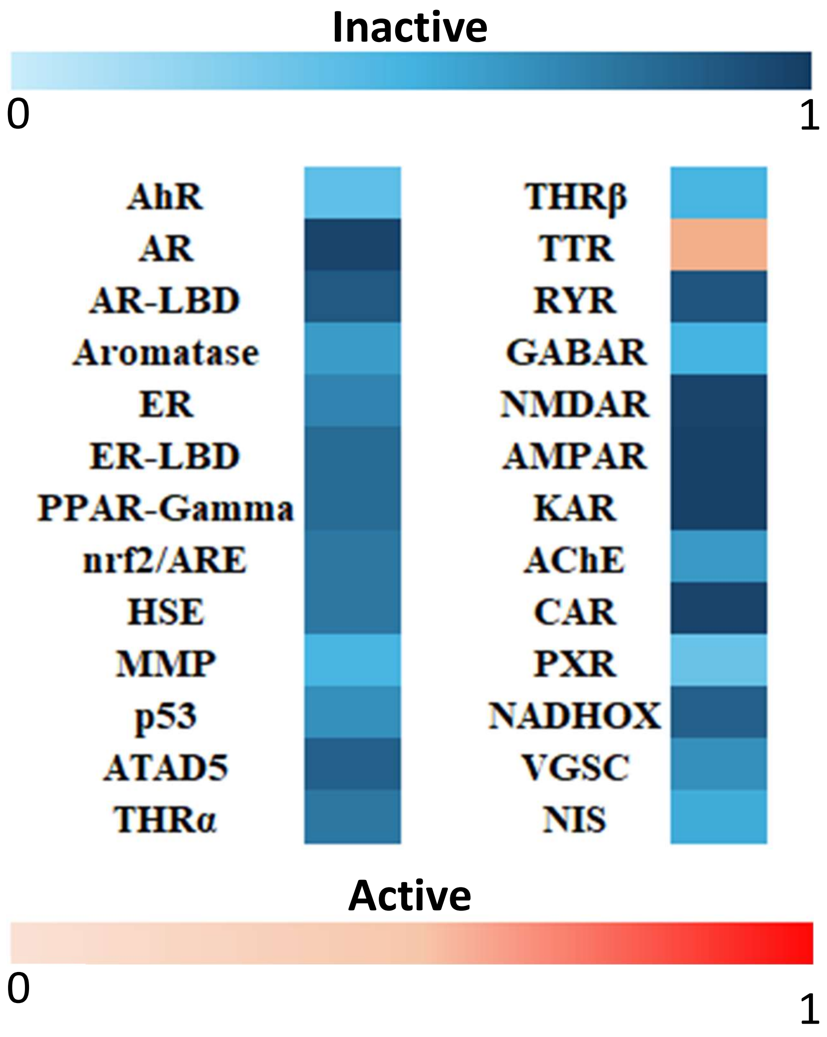

Figure S1. Heat map representing the activity of different biomarkers for toxicity pathways. The upper scale indicates inactivity levels (0 to 1, ranging from light blue to dark blue), while the lower scale represents activity levels (0 to 1, ranging from beige to red). Biomarkers are listed on the vertical axis, and their related intensities are shown in the corresponding columns. AhR: Aryl Hydrocarbon Receptor, AR: Androgen Receptor, AR-LBD: Androgen Receptor Ligand-Binding Domain, ER: Estrogen Receptor Alpha, ER-LBD: Estrogen Receptor Ligand-Binding Domain, PPAR-Gamma: Peroxisome Proliferator-Activated Receptor Gamma, nrf2/ARE: Nuclear Factor (Erythroid-Derived 2)-Like 2/Antioxidant Response Element, HSE: Heat Shock Factor Response Element, MMP: Mitochondrial Membrane Potential, p53: Phosphoprotein, ATAD5: ATPase Family AAA Domain-Containing Protein 5, THRα: Thyroid Hormone Receptor Alpha, THRβ: Thyroid Hormone Receptor Beta, TTR: Transthyretin, RYR: Ryanodine Receptor, GABAR: GABA Receptor, NMDAR: N-Methyl-D-Aspartate Glutamate Receptor, AMPAR: Alpha-Amino-3-Hydroxy-5-Methyl-4-Isoxazolepropionic Acid Receptor, KAR: Kainate Receptor, AChE: Acetylcholinesterase, CAR: Constitutive Androstane Receptor, PXR: Pregnane X Receptor, NADHOX: NADH-Quinone Oxidoreductase, VGSC: Voltage-Gated Sodium Channel, NIS: Na+/I− Symporter.
